# A Tale of Loops and Tails: The Role of Intrinsically Disordered Protein Regions in R-Loop Recognition and Phase Separation

**DOI:** 10.3389/fmolb.2021.691694

**Published:** 2021-06-10

**Authors:** Leonardo G. Dettori, Diego Torrejon, Arijita Chakraborty, Arijit Dutta, Mohamed Mohamed, Csaba Papp, Vladimir A. Kuznetsov, Patrick Sung, Wenyi Feng, Alaji Bah

**Affiliations:** ^1^ Department of Biochemistry and Molecular Biology, SUNY Upstate Medical University, Syracuse, NY, United States; ^2^ Department of Biochemistry and Structural Biology, University of Texas Health San Antonio, San Antonio, TX, United States; ^3^ Department of Urology, SUNY Upstate Medical University, Syracuse, NY, United States; ^4^ Bioinformatics Institute, A*STAR Biomedical Institutes, Singapore, Singapore

**Keywords:** R-loops, intrinscially disordered regions, R-loop interactome, liquid-liquid phase separation, R-loop readers, R-loop processing enzymes

## Abstract

R-loops are non-canonical, three-stranded nucleic acid structures composed of a DNA:RNA hybrid, a displaced single-stranded (ss)DNA, and a trailing ssRNA overhang. R-loops perform critical biological functions under both normal and disease conditions. To elucidate their cellular functions, we need to understand the mechanisms underlying R-loop formation, recognition, signaling, and resolution. Previous high-throughput screens identified multiple proteins that bind R-loops, with many of these proteins containing folded nucleic acid processing and binding domains that prevent (e.g., topoisomerases), resolve (e.g., helicases, nucleases), or recognize (e.g., KH, RRMs) R-loops. However, a significant number of these R-loop interacting Enzyme and Reader proteins also contain long stretches of intrinsically disordered regions (IDRs). The precise molecular and structural mechanisms by which the folded domains and IDRs synergize to recognize and process R-loops or modulate R-loop-mediated signaling have not been fully explored. While studying one such modular R-loop Reader, the Fragile X Protein (FMRP), we unexpectedly discovered that the C-terminal IDR (C-IDR) of FMRP is the predominant R-loop binding site, with the three N-terminal KH domains recognizing the trailing ssRNA overhang. Interestingly, the C-IDR of FMRP has recently been shown to undergo spontaneous Liquid-Liquid Phase Separation (LLPS) assembly by itself or in complex with another non-canonical nucleic acid structure, RNA G-quadruplex. Furthermore, we have recently shown that FMRP can suppress persistent R-loops that form during transcription, a process that is also enhanced by LLPS *via* the assembly of membraneless transcription factories. These exciting findings prompted us to explore the role of IDRs in R-loop processing and signaling proteins through a comprehensive bioinformatics and computational biology study. Here, we evaluated IDR prevalence, sequence composition and LLPS propensity for the known R-loop interactome. We observed that, like FMRP, the majority of the R-loop interactome, especially Readers, contains long IDRs that are highly enriched in low complexity sequences with biased amino acid composition, suggesting that these IDRs could directly interact with R-loops, rather than being “mere flexible linkers” connecting the “functional folded enzyme or binding domains”. Furthermore, our analysis shows that several proteins in the R-loop interactome are either predicted to or have been experimentally demonstrated to undergo LLPS or are known to be associated with phase separated membraneless organelles. Thus, our overall results present a thought-provoking hypothesis that IDRs in the R-loop interactome can provide a functional link between R-loop recognition *via* direct binding and downstream signaling through the assembly of LLPS-mediated membrane-less R-loop foci. The absence or dysregulation of the function of IDR-enriched R-loop interactors can potentially lead to severe genomic defects, such as the widespread R-loop-mediated DNA double strand breaks that we recently observed in Fragile X patient-derived cells.

## Introduction

Co-transcriptional R-loops are widespread and functional non-canonical nucleic acid structures (Santos-Pereira and Aguilera, 2015; Crossley et al., 2019; García-Muse and Aguilera, 2019; Hegazy et al., 2020). In mammalian cells, for instance, R-loops occupy as much as 5% of the genome, usually at promoter and terminator regions as well as at ribosomal DNA and transfer RNA gene regions (Sanz et al., 2016). R-loop forming sequences (RLFS) are included in more than 75% of annotated genes (Wongsurawat et al., 2012; Jenjaroenpun et al., 2015; Jenjaroenpun et al., 2017), and detail information about genome-wide experimental and computationally predicted R-loops (including RLFS and R-loop boundaries) is presented in an R-loop database named R-loopDB (Jenjaroenpun et al., 2017). Elucidating the biological functions of R loops is an active area of research as dysregulation of R-loop function is linked to many diseases, such as cancer and neurological disorders (Wongsurawat et al., 2012; Kuznetsov et al., 2018; De Magis et al., 2019; Perego et al., 2019). Thus, understanding the mechanisms of R-loop formation and interaction, and the processes that regulate or are regulated by R-loops is an important first step for determining the cellular functions of R-loops. Furthermore, unravelling the structural and binding mechanisms utilized by proteins that are involved in the regulation of R-loop formation, prevention and resolution, as well as understanding how these cellular processes are dysregulated in pathological conditions, is vital for developing novel therapeutics to target the biological functions of R-loops.

Functionally, R-loops have been implicated in several biological processes including, but not limited to: 1) class switch recombination in B cells (Yu et al., 2003; Ribeiro de Almeida et al., 2018), 2) replication in bacterial (Kogoma, 1997), mitochondrial (Xu and Clayton, 1996; Pohjoismäki et al., 2010), and Bacteriophage T4 (Kreuzer and Brister, 2010) DNA, 3) telomere lengthening (Balk et al., 2013; Pfeiffer et al., 2013), 4) faithful chromosome segregation (Kabeche et al., 2018), 5) transcription regulation and gene expression (Wongsurawat et al., 2012; Ginno et al., 2013; Skourti-Stathaki and Proudfoot, 2014; Sanz et al., 2016; Kuznetsov et al., 2018), 6) DNA repair (Ohle et al., 2016; Lu et al., 2018), 7) chromatin opening, 8) cell proliferation (Yeo et al., 2014) and 9) cell differentiation (Wongsurawat et al., 2012; Kuznetsov et al., 2018; García-Muse and Aguilera, 2019). Nevertheless, R-loops are also a known source of genomic instability (Aguilera and García-Muse, 2012; Skourti-Stathaki and Proudfoot, 2014; Sollier et al., 2014; Sollier and Cimprich, 2015; Costantino and Koshland, 2018; Crossley et al., 2019; Hegazy et al., 2020), including 1) DNA strand breaks (Wimberly et al., 2013; Cristini et al., 2019) 2) mutations (Muramatsu et al., 2000; Wimberly et al., 2013) 3) recombination (Gan et al., 2011; Alzu et al., 2012) and 4) chromosome rearrangements (Chiarle et al., 2011; Costantino and Koshland, 2018; So and Martin, 2019) leading to cancer (Boros-Oláh et al., 2019; Crossley et al., 2019; De Magis et al., 2019) and neurological disorders (Wongsurawat et al., 2012; Groh et al., 2014; Kuznetsov et al., 2018). Thus, balancing the biological functions of R-loops is important for regulating genome stability, transcription, and gene expression through a variety of genetic and epigenetic regulatory mechanisms (Skourti-Stathaki et al., 2011; Ginno et al., 2012; Castellano-Pozo et al., 2013; Ginno et al., 2013; Skourti-Stathaki et al., 2014; Boque-Sastre et al., 2015; Sanz et al., 2016). Tight regulation of R-loop formation, signaling and resolution, along with regulation of the functions of proteins involved in these processes, are of utmost importance in order to maintain the physiological roles of R-loops. The absence or dysfunctions of these regulatory mechanisms will result in deleterious consequences such as genome instability, potentially leading to devastating diseases (Salvi and Mekhail, 2015; Perego et al., 2019). Thus, it is critically important to elucidate how R-loop recognition, signaling and resolution are mediated in normal and pathological conditions. While studying the impact on genome stability due to the absence of FMRP in Fragile X patient-derived (FX) cells, we discovered that FX cells undergo R-loop-mediated genome-wide DNA double-strand breaks (DSBs) under aphidicolin-induced DNA replication stress (Chakraborty et al., 2020). We subsequently demonstrated that FMRP directly interacts with R-loops, predominantly *via* its C-IDR, with the three N-terminal folded RNA binding KH domains providing additional weak contacts through binding to the various R-loop substructures (Chakraborty et al., 2021). This surprising and exciting finding prompted us to investigate the role of IDRs in the other R-loop interacting proteins, especially in proteins that lack the canonical R-loop processing enzyme domains such as helicases or nucleases.

Herein, we perform an integrated bioinformatics and computational biology study by evaluating IDR prevalence and LLPS propensity as well as by analyzing amino acid sequence composition of the IDRs in the R-loop and DNA:RNA hybrid interactomes. Our goal is to elucidate the types of physical interactions and chemical properties enabling potential IDR-mediated R-loop recognition, signaling, and assembly. We found that ∼66% of the combined R-loop and DNA:RNA hybrid interactomes contain at least one IDR with 30 or more consecutive residues, with the average IDR content being ∼29 ± 26% of the total protein length. However, when we considered the set of R-loop Readers with RRM or KH domains, we found that ∼87% of these Readers contain at least one IDR with 30 or more consecutive residues, with the average IDR content being ∼48 ± 25%. In contrast, for the set of R-loop Enzymes with helicase or hydrolase activity, only ∼66% contain at least one IDR with 30 or more consecutive residues, with the average IDR content being ∼18 ± 15%. We also found that the IDRs of the R-loop interactome contain low complexity sequences with heavy biases towards a few residues (Glu, Ser, Lys, Pro, Gly, Ala, and Arg), with the IDRs of the R-loop Readers being enriched in Gly, Ser, Arg, and Pro residues and the IDRs of the Enzymes enriched in Glu, Lys, Arg, and Ser. However, these differentially biased amino acid compositions become more striking when we analyze the 2-mer (dipeptides) and 3-mer (tripeptides) compositions and patterns found in the IDRs of Readers vs. Enzymes. For instance, we found that the most prevalent dipeptides are GG, PP, RS, SR, and RG for the R-loop Readers, and EE, KK, KE, EK, and GG for the R-loop Enzymes. Indeed, we observed even more pronounced differences from the 3-mer analysis, where the most frequent tripeptides are GGG, SRS, RSR, PPP, and GRG for the Readers, but EEE, GGG, KKK, KEE, and EKE for the Enzymes. Thus, the 2-and 3-mer amino acid composition and sequence patterning in the IDRs of R-loop Readers are more similar to the C-IDR of FMRP than those in the IDRs of the Enzymes. This finding suggests that R-loop Readers may potentially interact with R-loops using a mechanism similar to that of the C-IDR of FMRP. Finally, using two LLPS predictors, catGRANULE (Bolognesi et al., 2016) and PScore (Vernon et al., 2018), we show that ∼67 and ∼59% of Readers were predicted to undergo LLPS by catGRANULE and PScore analyses, respectively while for the Enzymes, it was only ∼31 and ∼17%, respectively. However, when we analyzed the LLPS databases including PhaSePro (Mészáros et al., 2020), LLPSDB (Li et al., 2020), and DRLLPS (Ning et al., 2020), up to ∼89 and ∼83% of Readers and Enzymes, respectively, were found to localize to or associate with phase-separated membraneless cellular organelles, suggesting that unlike Readers that can potentially act as scaffolds, Enzymes can be recruited to these organelles as clients. Thus, the presence of low complexity sequences in the IDRs of R-loop Readers, as well as the modular domain architecture of the R-loop interactome, can provide a functional link between R-loop recognition and downstream signaling/processing through the assembly of membraneless R-loop foci. Inside these foci, the physiological/pathological roles of these intricate nucleic acid structures, mediated by the synergy between the IDRs and the folded domains of the Readers as well as the activities of the Enzymes, can be coordinated.

## Results

### Structural Mechanism of R-Loop Recognition

**FIGURE 1:**
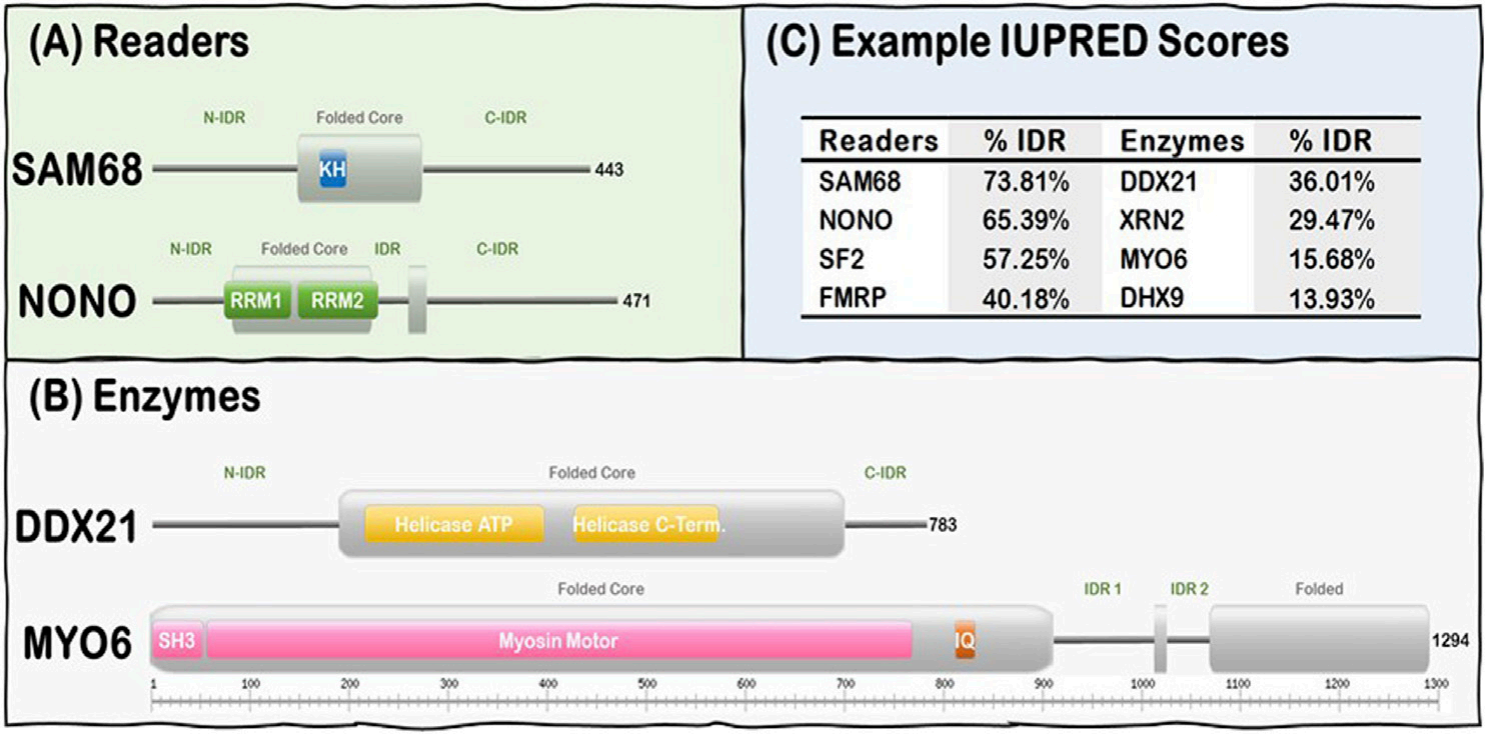
R-loop interactome proteins are modular, containing both folded domains, and IDRs. **(A)** Examples of Readers containing KH domains (SAM68) and RRM domains (NONO). **(B)** Examples of Enzymes from the Helicase (DDX21) and the Hydrolase (MYO6) subgroups. Note Folded (gray rectangles) and IDRs (gray lines) are predicted from our IUPRED results while domain localization (e.g., KH, RRM, and SH3) was obtained from the UniProt database (UniProt: The universal protein knowledgebase in 2021, 2021). **(C)** Representative Readers and Enzymes with their IDR content (%).

R-loop resolving enzymes, such as topoisomerases, nucleases and helicases, have specialized folded catalytic domains that allow them to perform their biological functions and thus mitigate the deleterious effects of dysregulated R-loop formation. For instance, topoisomerases, such as Top1, are known to prevent R-loop formation during transcription by reducing the negative supercoil formed behind RNA Pol II (Tuduri et al., 2009; El Hage et al., 2010; Marinello et al., 2016), while helicases act downstream to promote R-loop resolution by unwinding the DNA:RNA duplex as described for Senataxin (Skourti-Stathaki et al., 2011; Yeo et al., 2014; Cohen et al., 2018), DDX5 (Mersaoui et al., 2019), and Aquarius (De et al., 2015; Sakasai et al., 2017). Nucleases, such as RNAse H1, act downstream to promote R-loop resolution by digesting the hybridized RNA from the DNA:RNA duplex (Keller and Crouch, 1972; Wahba et al., 2011). In contrast, R-loop Readers can act downstream to promote R-loop resolution by linking their ability to bind R-loops with their capacity to recruit other factors to ultimately resolve the formed R-loops in a timely manner (Arab et al., 2019). Intriguingly, R-loop Enzymes and Readers are modular proteins that contain both folded domains and long stretches of IDRs (Figure 1). These domains are usually nucleic acid processing (e.g., nucleases, helicases) and binding (e.g., RRMs, KH) modules. However, there is currently no well-established general mechanism of how the IDRs of the R-loop interactome synergize with their folded domain counterparts in R-loop recognition, signaling, and resolution.

**FIGURE 2:**
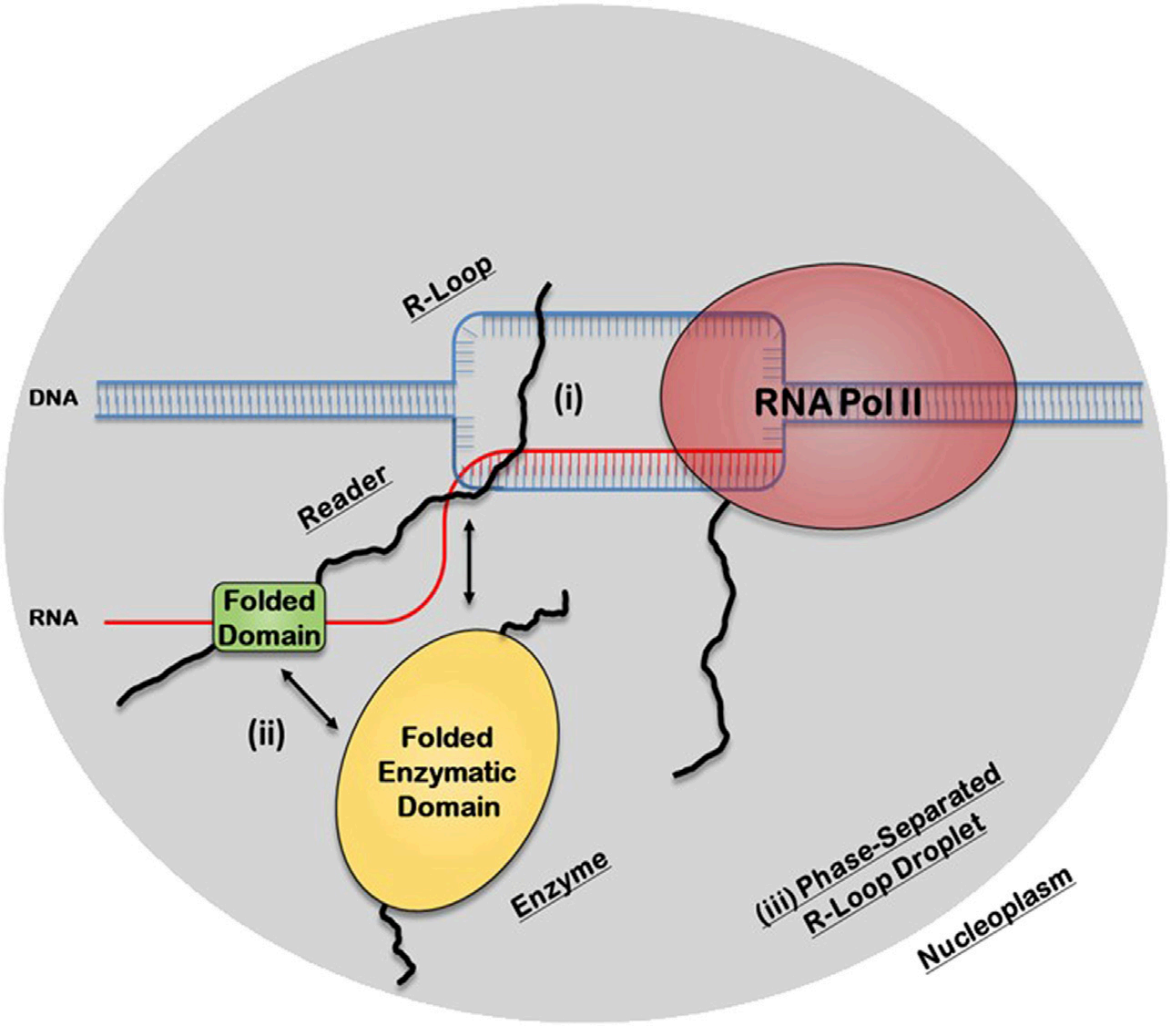
Potential structural mechanisms by which IDRs and folded domains of R-loop proteins can synergize to regulate the biological functions of R-loops. In the first mechanism (i), the IDR is directly involved in the recognition of the R-loop structure itself, while in the second mechanism (ii), the folded domain does the binding and the IDR is involved in recruiting other R-loop processing factors. In the third mechanism (iii), the IDR, in concert with the folded domains and the R-loop structure itself, can mediate the assembly of membraneless R-loop processing and signaling foci *via* transient multivalent intermolecular interactions.

There are three potential structural mechanisms by which this synergy afforded by the IDRs can occur to mediate the biological functions of R-loops (Figure 2). IDRs can be involved in 1) direct recognition of the R-loop structure itself 2) recruitment of other R-loop processing factors or 3) assembly of membraneless R-loop foci. In the first synergistic mechanism, the IDRs can be involved in the direct interaction with the R-loops *via* 1) binding to individual segments of the R-loop sub-structure, such as the dsDNA, displaced ssDNA, trailing ssRNA overhang, branching of the dsDNA, or the DNA:RNA hybrid; 2) binding a unique structural feature that emerges from the distinct 3D architecture of the R-loop structure, such as the *junction* where the dsDNA, the ssDNA, and the DNA:RNA hybrid all intersect or the local 3D structure formed by the triple stranded structure of the R-loop (i.e., DNA:RNA-ssDNA sub-structure); or 3) binding the entire 3D R-loop structure as a unit. Interestingly, these recognition mechanisms can potentially be mediated by stable complexes due to disorder-to-order transitions that IDRs normally undergo or by formation of dynamic “fuzzy” IDR:nucleic acid complexes. In the second mechanism, upon binding of the R-loop structure by the folded domain, the IDRs can recruit other partners through protein-protein interactions to the site of R-loop formation. In the third mechanism, IDRs within the R-loop interactome can mediate the assembly of membraneless R-loop foci *via* LLPS. As described below, this process will generate a microenvironment conducive for R-loop processing and signaling. Finally, these three mechanisms are non-exclusive and could simultaneously occur and complement each other.

Moreover, synergistic R-loop interactions can occur in a single polypeptide chain or in two or more protomers in a homo/hetero-complex. For example, it has been previously demonstrated that GADD45A, a monomeric protein, binds R-loops by directly interacting with the DNA:RNA hybrid structure without interacting with the ssRNA, ssDNA, or dsDNA (Arab et al., 2019). In contrast, the ALBA proteins bind R-loops as a heterodimer, whereby the ALBA1 and ALBA2 protomers bind the DNA:RNA hybrid and the ssDNA, respectively (Yuan and et al., 2019). Furthermore, in the case of the FANCI-FANCD2 heterodimer, the complex binds R-Loops *via* interaction with the displaced ssDNA strand and ssRNA tail (Liang et al., 2019). In all these cases described so far, it is the folded nucleic acid binding domains of the R-loop Readers that are responsible for directly binding the R-loop structure, with the IDR playing other, albeit important, roles in the formation of the protein:R-loop complex. However, given the fact that IDRs play important roles in directly recognizing DNA, RNA, and other non-canonical complex nucleic acid structures like G-quadruplexes (Fuxreiter et al., 2011; Brázda et al., 2014), a fascinating question is whether the IDRs, rather than the folded domains, of modular R-loop interactors can be the predominant site for recognizing R-loops. In fact, there are numerous instances of IDRs of proteins playing critical roles in the biological functions of many proteins due to their inherent ability to form flexible linkers between folded domains, for being the predominant sites for post translational modifications (PTMs) and for serving as sites for direct protein and nucleic acid binding as well as for being the dominant drivers of LLPS for many known phase separating proteins (Van Der Lee et al., 2014; Uversky, 2017). In this manuscript, we investigate the amino acid composition and properties of the proteins in the R-loop interactome and explore the hypothesis that, in some R-loop binding proteins, the IDRs provide the dominant site of interaction with the R-loop structure as we recently demonstrated for FMRP (Chakraborty et al., 2021).

### The C-IDR of the Fragile X Syndrome Protein (FMRP): A Canonical Intrinsically Disordered Region R-Loop Reader

**FIGURE 3:**
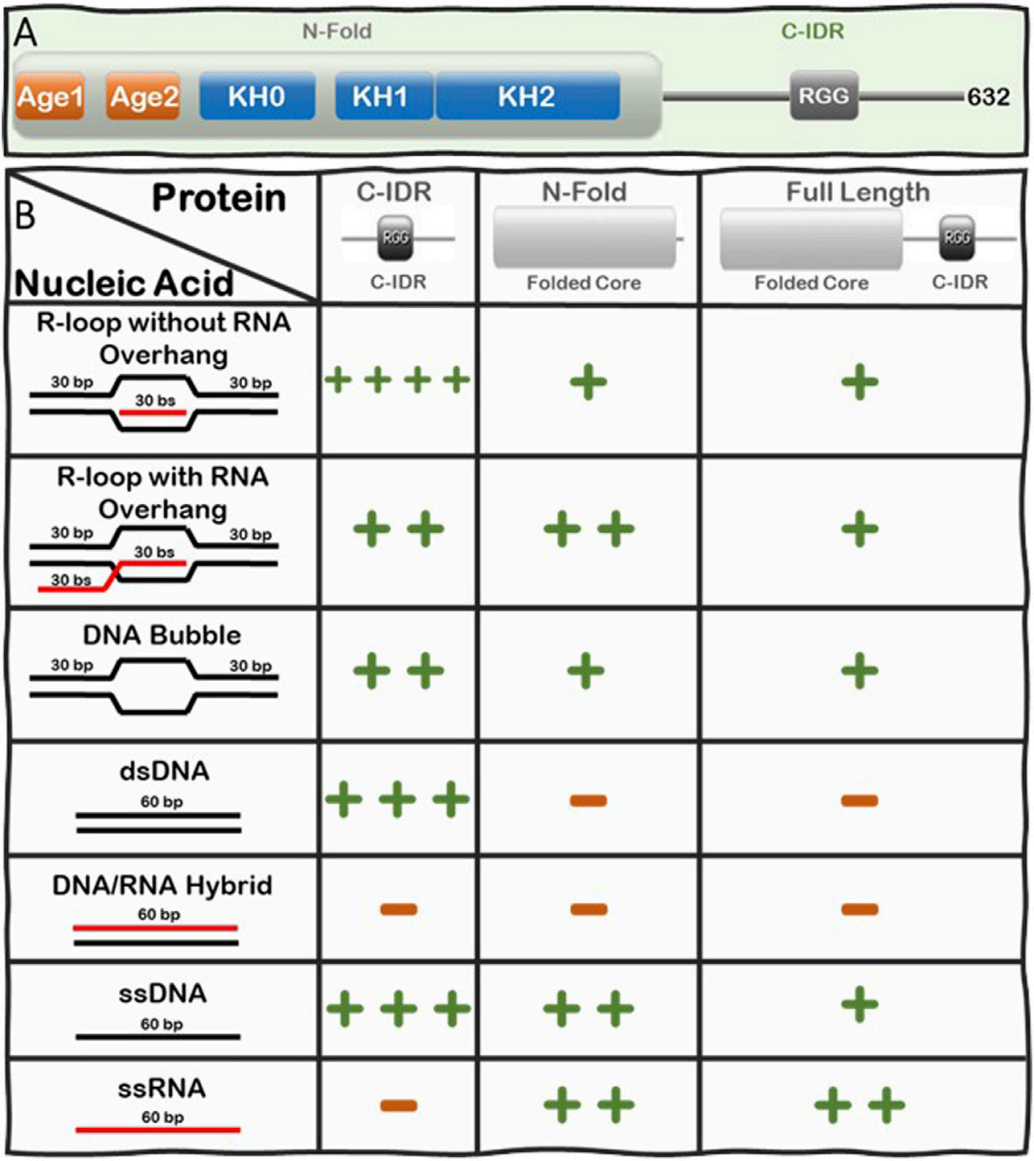
Summary of the Interactions of FMRP full-length, N-Fold, and C-IDR with R-loops and substructures. **(A)** Modular domain architecture of FMRP showing the N-terminal folded core (N-Fold), consisting of two Agenet domains and three KH domains, and the C-terminal intrinsically disordered region (C-IDR). **(B)** Table showing the relative strength of binding between FMRP constructs (top row) and various nucleic acid substrate controls (left column), such as R-loops with (out) RNA overhang, DNA bubble, dsDNA, DNA:RNA hybrid, ssDNA, and ssRNA. Binding affinity for each FMRP construct and nucleic acid substrates was calculated as dissociation constants (K_D_s) averaged from at least two Electrophoretic Mobility Shift Assay (EMSA) experiments(Chakraborty et al., 2021). K_D_ values are scaled according to a log scale: (++++) means 1–10 nM; (+++) means 11–100 nM; (++) means 0.1–1 μM; (+) means 1–10 μM; and (−) means no observed interaction under the binding conditions used for the EMSA assay (i.e., 1 nM of γ-P (Muramatsu et al., 2000) R-loop, RNA-DNA hybrid, dsDNA, bubble DNA, ssDNA, or RNA substrate mixed with protein at various concentrations in a buffer composed of 25 mM Tris-HCl (pH 7.5), 100 mM KCl, 5 μg/ml BSA, and 5 mM EDTA. Details of the original SDS PAGE gels of the protein constructs and the phosphorimages of the EMSA gels, data quantification, binding curves where the K_D_s are extracted are all shown in Figure 2 and Supplementary Figure S2 in ref (Chakraborty et al., 2021).

Loss of function or lack of expression of FMRP causes Fragile X Syndrome (FXS), a neurodevelopmental disease that results in learning disabilities and cognitive impairment (Ashley et al., 1993; Brown et al., 2001; Garber et al., 2008; Santoro et al., 2012). We recently discovered there is a significant increase in R-loop-mediated DNA double strand breaks (DSBs) in FXS patient-derived lymphoblastoids compared to control cells (Chakraborty et al., 2020) and therefore, we were interested in understanding the underlying mechanism driving this observation. To elucidate this mechanism, we set out to investigate whether FMRP interacts with R-loops directly or indirectly. We tested the binding of full-length and fragments of FMRP to an array of nucleic acid substrates including R-loops with and without RNA overhangs using Electrophoretic Mobility Shift Assays (EMSA). FMRP is a multi-functional modular protein consisting of an N-terminal folded core (N-Fold) and a long C-IDR (Figure 3A). The N-Fold of FMRP contains two methylated arginine-binding Agenet domains (Myrick et al., 2015) and three RNA-binding KH domains (Valverde et al., 2007; Myrick et al., 2015), which are required for binding chromatin (Alpatov et al., 2014) and for interacting with RNA substrates (Santoro et al., 2012), respectively. Interestingly, KH domains are known to interact with various types of nucleic acids (e.g., ssRNA, ssDNA, and dsDNA) (Valverde et al., 2008), while the C-IDR of FMRP is well known for binding various mRNA substrates with intricate secondary/tertiary structures such as G-quadruplexes (Vasilyev et al., 2015; Hänsel-Hertsch et al., 2017) and SoSLIP (Sod1 Stem Loops Interacting with FMRP) (Blackwell et al., 2010; Bechara and et al., 2009).

We systematically tested the ability of FMRP full-length, N-Fold and C-IDR to bind nucleic acid structures including ssDNA, dsDNA, RNA, DNA:RNA hybrid, and R-loops with and without RNA overhang (Figure 3B). Indeed, we observed direct binding between FMRP full-length and R-loops and, of all the tested protein-nucleic acid pairs, the C-IDR and the R-loop substrate without overhang produced the highest affinity (K_D_ = 4.73 ± 3.83 nM) (Chakraborty et al., 2021). However, the interaction was significantly weakened when a 5’ RNA overhang was present in the R-loop (K_D_ = 148.3 ± 10.03 nM), suggesting that the C-IDR may interact with the triple junction where the trailing RNA emerges. Furthermore, while the C-IDR showed affinity towards ssDNA and dsDNA in isolation, it barely interacted with the DNA:RNA hybrid or ssRNA (Note: our RNA does not contain consensus FMRP binding sites). The fact that the C-IDR binds more tightly to the R-loop substrate compared to the ssDNA or the dsDNA, and that it does not bind the DNA:RNA hybrid control, suggests that the C-IDR specifically interacts with R-loops through binding to a distinct 3D architectural feature of the R-loop structure *via* multiple interfaces and that the RNA overhang interferes with this interaction. In contrast, the N-Fold binds R-loops with ssRNA overhang tighter (K_D_ = 320 ± 3.03 nM) compared to the other substrates tested, but its affinity towards the R-loop substrates are significantly lower compared to that of the C-IDR (i.e., 320 ± 3.03 nM vs 4.73 ± 3.83 nM). Furthermore, N-Fold shows affinities for ssRNA and ssDNA, but not for dsDNA nor the DNA:RNA hybrid. Therefore, the N-Fold likely interacts with the R-loop through binding to the single stranded segments (RNA or DNA) of the R-loop using its KH domains. Taken together, these data suggest that 1) there are multivalent interactions with various affinities between the different segments of FMRP and the various substructures of R-loops 2) the C-IDR is the predominant region that interacts with R-loop substrates without 5’-RNA overhangs and 3) the KH domains in the N-Fold prefer R-loops with a 5’ RNA overhang.

Finally, we subsequently showed that FMRP co-immunoprecipitates with DHX9 *in vivo* and directly binds the methylated Arginine residues in the RGG region of DHX9 *in vitro,* using the two Agenet domains within the N-Fold (Chakraborty et al., 2021). DHX9 is a multifunctional ATP-dependent nucleic acid helicase that unwinds various DNA and RNA substrate structures, including R-loops and G-quadruplexes (Chakraborty and Grosse, 2011). Thus, our data suggests that the multi-domain FMRP bridges the interaction between R-loops and R-loop resolvases through its C-IDR and N-Fold, respectively. Therefore, we wondered whether this or a similar mechanism of R-loop recognition and recruitment of R-loop resolvases is unique to FMRP or universal to all R-loop Readers. To explore this question, we compare and contrast the similarities and differences between the physico-chemical properties of the C-IDR of FMRP and the IDRs of other proteins in the known R-loop interactome.

### Classifying the R-Loop Interactome

**FIGURE 4:**
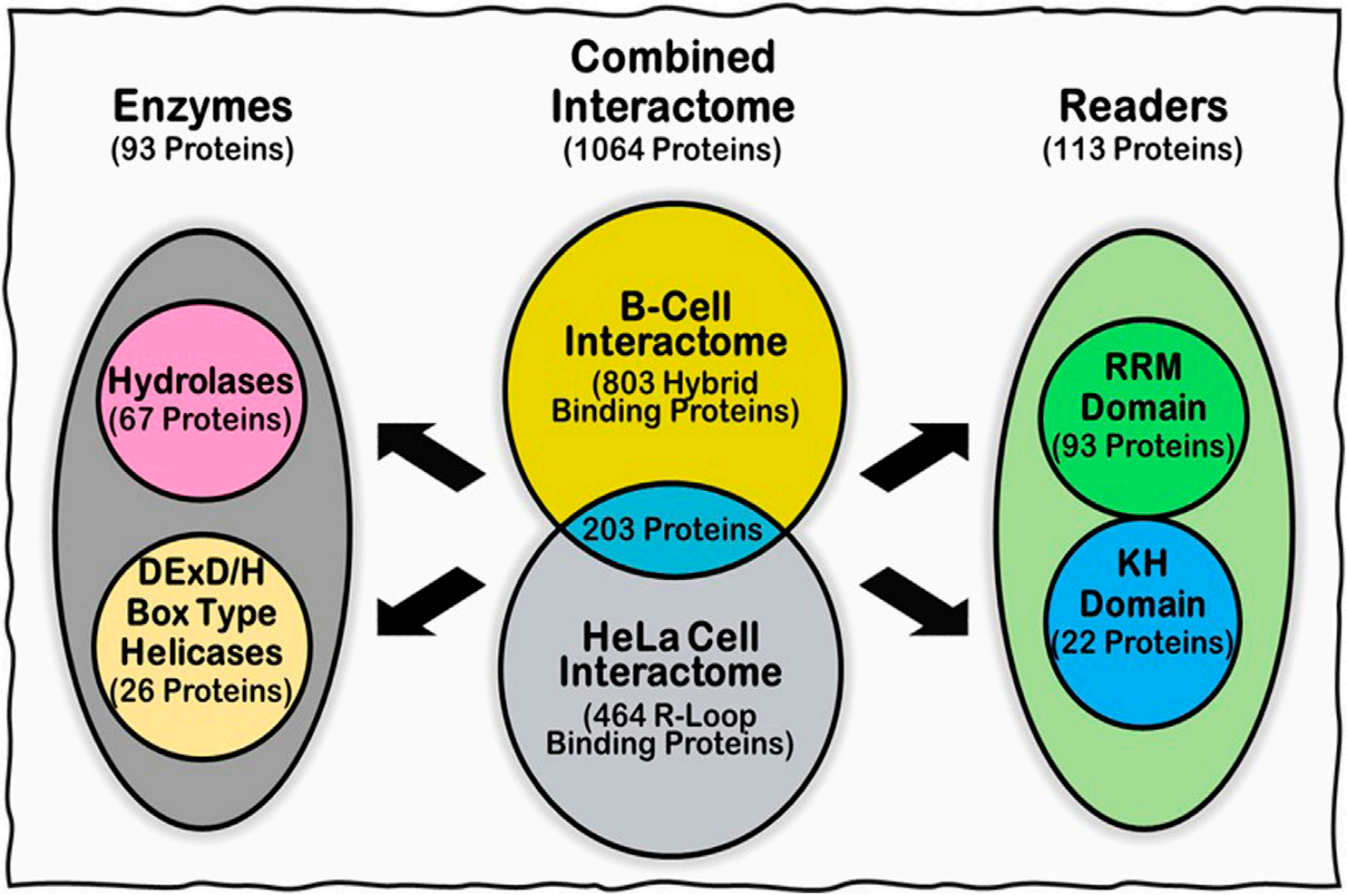
Summary of the R-loop interactome and the Subgroups (Readers and Enzymes), whose IDRs were analyzed in this work. We focused on investigating the IDRs of proteins from three major groups: (i) Combined R-loop and DNA:RNA hybrid Interactomes from the B-Cell (803 proteins) and HeLa Cell (464 proteins) studies, respectively, not including multiple isoforms from the same protein; (ii) Readers: comprised of 22 KH domain- and 93 RRM-containing proteins from the Combined Interactomes (with two proteins containing both KH and RRM domains); and (iii) Enzymes: comprised of 26 DEAD/DEAH box type DNA:RNA helicases and the 67 P-loop triphosphate hydrolases identified in the B-Cell interactome. For more details, please see text (Wang et al., 2018).

A myriad of proteins with different functions are responsible for regulating the formation, signaling and timely resolution of R-loops. Many R-loop interacting proteins were identified in the literature over the last few decades (Li and Manley, 2005; Cerritelli and Crouch Ribonuclease, 2009; Tuduri et al., 2009; El Hage et al., 2010; Skourti-Stathaki et al., 2011; Herrera-Moyano et al., 2014), but no large-scale investigation in human cells was conducted until 2018. That year, Cristini et al. (2018) analyzed the R-loop interactome in HeLa cells and, later in the same year, Wang et al. (2018) analyzed the DNA:RNA hybrid interactome in human B-cells. Cristini et al. (2018) used affinity purification with the S9.6 antibody followed by mass spectrometry (MS) analysis, while Wang et al. (2018) conducted pull-down assays using synthetic versions of two different DNA:RNA hybrids from canonical R-loop sequences found in the BAMBI and the DPP9 genes, followed by MS to isolate the interacting proteins. While these studies missed some key R-loop proteins, they recovered most proteins traditionally known to be involved with R-loop regulation, such as topoisomerases [e.g., Top1 (Tuduri et al., 2009; El Hage et al., 2010)] and nucleases [e.g., XRN2 (Morales et al., 2016)] as well as new candidate proteins that were never reported to be involved in R-loop biology. In the HeLa cell R-loop interactome, a total of 464 R-loop interacting proteins were identified with a high enrichment of RNA and DNA binding domains (38 and 15%, respectively), followed by mRNA/rRNA processing factors, DNA and RNA helicases, nucleases and chromatin proteins. Interestingly, the authors compared the HeLa cell R-loop interactome with the HeLa cell mRNA interactome and, despite a large overlap between the two proteomes (i.e., 287 proteins), a significant part of the R-loop interactome (i.e., 187 proteins) is unique. In contrast, for their B-Cell interactome studies, (Wang et al., 2018) identified a total of 803 proteins that could bind their bait DNA:RNA hybrid sequences. The resulting hybrid interactome is highly enriched for proteins involved in RNA binding, mRNA splicing, ATP-dependent helicase activity, termination of RNA pol II transcription, regulation of telomerase, and RNA localization to Cajal Body. When the authors searched for domains present in the B-Cell DNA:RNA hybrid interactome, they found five highly enriched functional domains including alpha-beta plait domains, DEAD/DEAH box type DNA/RNA helicase domains, KH domains, P-loop triphosphate hydrolase, and OB-fold domains. When the two R-loop interactomes were compared, 203 overlapping proteins were identified between the two studies (Figure 4). Thus, there are plenty of high confidence candidates from these high-throughput studies, as well as from other previously identified bona fide R-loop binding proteins from other organisms, for investigating the role of IDRs in the mechanism of R-loop recognition, processing and resolution.

For this study, we focused on investigating the IDRs of proteins from three major groups (Figure 4): 1) Combined Interactome: comprised R-loop and DNA:RNA hybrid Interactomes from the B-Cell (Wang et al., 2018) and HeLa Cell (Cristini et al., 2018) studies respectively; 2) Readers: comprised of only KH domain-and RRM-containing proteins from the Combined interactomes; and 3) Enzymes: comprised of the DEAD/DEAH box type DNA:RNA helicases and the P-loop triphosphate hydrolases identified in the B-Cell interactome (Figure 4). In the following sections, we investigate the prevalence of IDRs, the propensity for LLPS, and the properties of the amino acid composition for these groups of R-loop associated proteins.

### Prevalence of IDRs in the R-Loop and DNA:RNA Interactomes

The discovery of intrinsically disordered proteins (IDPs) and IDRs upends the traditional structure-function paradigm, which states that the biological function of a protein depends on its ability to fold into a well-defined 3D-structure (Dyson and Wright, 2005). ∼30% of eukaryotic proteins are predicted to be entirely disordered or to contain long stretches of disordered residues (Van Der Lee et al., 2014). It is now generally accepted that proteins can exist and be fully functional in a continuum of structural and dynamic states, ranging from stably folded to completely disordered states. Unlike folded domains that exist in one or few stable conformations, IDRs consist of an ensemble of rapidly interconverting conformations, which play critical roles in diverse biological processes including cell signaling and cell cycle regulation, mRNA translation and splicing as well as DNA replication and transcription-all processes that are often dysregulated in many human diseases (Uversky et al., 2008; Babu et al., 2011; Anbo et al., 2019). Interestingly, most proteins are modular with a mix of both folded domains and IDRs, thus providing an intramolecular synergy that significantly expands their functional repertoire (Babu et al., 2012).

**FIGURE 5:**
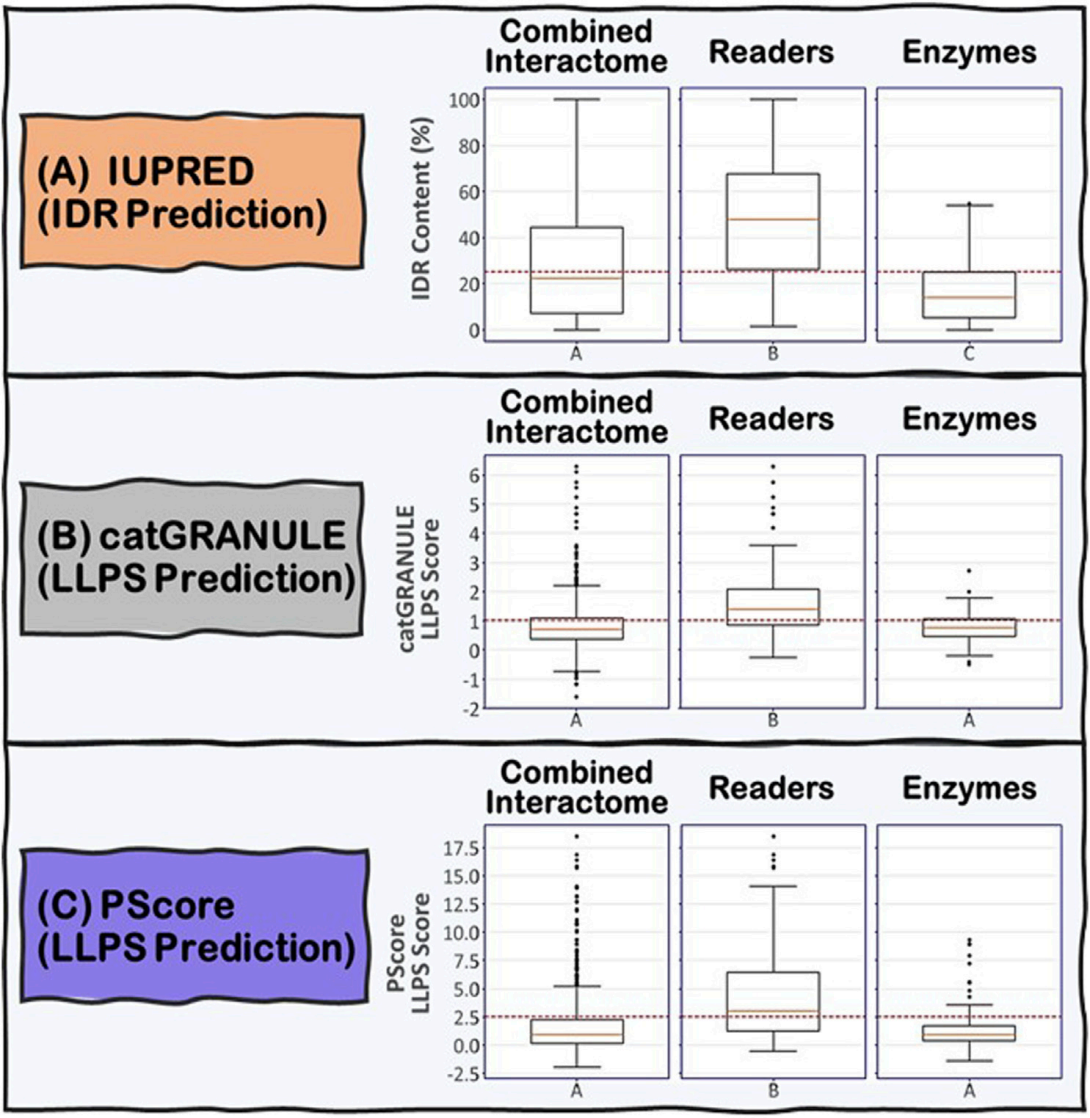
Boxplots showing the distribution of **(A)** IDR content and **(B)** and **(C)** LLPS propensity predictions for the Combined Interactome, Readers and Enzymes used in this study. **(A)** IDR content predicted by IUPRED indicates that the three groups are significantly different (*p* < 0.05) with the Readers possessing the highest IDR content. **(B)** and **(C)** LLPS propensity predicted by catGRANULE and PScore indicate that the Readers are significantly different from the other two groups (*p* < 0.05) possessing the highest LLPS propensity while Enzymes and Combined Interactome are not significantly different from each other (*p* < 0.05). The established critical threshold for each prediction is represented in dashed red line: **(A)** 25% IDR content, **(B)** 1.0 LLPS propensity score, **(C)** 2.5 LLPS propensity score. This critical threshold suggests whether a protein possesses **(A)** substantial IDR content and **(B)** and **(C)** potential to promote LLPS.

To address the role of IDRs in the proteins known to be involved in R-loop biology, we undertook an integrated computational biology and bioinformatics approach. For each protein in the combined R-loop and DNA:RNA hybrid interactomes, we identified and extracted their IDRs to analyze the amino acid composition, physico-chemical properties and molecular features present in these sequences. The IDRs were predicted using the IUPRED program (Erdős and Dosztányi, 2020), whose algorithm predicts disordered regions by estimating their total pairwise inter-residue interaction energy, assuming that IDRs do not fold due to their inability to form sufficient stabilizing inter-residue interactions. The program is also optimized for predicting short or long disordered regions and structured domains. Initially, we defined an IDR as a protein segment with at least 30 consecutive amino acids with a predicted IUPRED disordered score greater than or equal to 0.5, with a tolerance for stretches of at most 10 amino acids whose score is less than 0.5 within the IDR. This process is monitored by an integrated confidence score that decreases for each exceptional amino acid within the IDR. We have also tested IDRs with at least 20 or at least 40 consecutive amino acids long (Supplementary Table S1A). In summary, we found that 74, 66 and 59% of the proteins in the combined interactome contained at least one IDR of at least 20, 30, and 40 residues long, respectively, with the overall average fraction of intrinsic disordered residues in these proteins being ∼29% with a standard deviation of ∼26%. However, when we examined the Readers, we found that 91, 87, and 81% of the Readers contain at least one IDR of at least 20, 30, and 40 residues long, respectively, and the overall average of intrinsic disorder in the Readers increased to ∼48% with a standard deviation of ∼25%. In contrast, for the Enzymes, the percentage of proteins with at least one IDR of at least 20, 30, and 40 residues long are 73, 66, and 59%, respectively, with the overall average of intrinsic disorder in the Enzymes being ∼17% with a standard deviation of ∼14%. Taken together, these data suggest that IDRs are more prevalent and form a greater integral component in the modular domain organization of Readers than in Enzymes (Figure 5A).

### Propensity of the R-Loop Interactome to Undergo LLPS

Next, we examined the propensity for the R-loop interactome proteins to phase separate. LLPS is increasingly being recognized as a key organizing principle of eukaryotic nuclei (Zhu and Brangwynne, 2015; Strom and Brangwynne, 2019; Sabari et al., 2020). The nucleoplasm contains a large number of LLPS-driven membraneless organelles (MLOs), including the nucleolus, Cajal bodies, Histone Locus Body, transcription factories, DNA repair foci, paraspeckles, nuclear speckles, and PML bodies (Zhu and Brangwynne, 2015). These MLOs form at specific sites to influence gene expression, for example, by enhancing the transcription of specific cluster of genes (e.g., rDNA and histone genes in the nucleolus and Histone Locus Body respectively), RNA splicing (e.g., Nuclear Speckles) and the expression and processing of small nuclear and nucleolar RNAs (sn/snoRNAs) in Cajal bodies. Since R-loop formation is directly coupled to transcription, which has been shown to be enhanced by LLPS *via* the assembly of membraneless transcription factories (Boija et al., 2018; Sabari et al., 2018), and because R-loop Readers like FMRP have been previously shown to undergo LLPs with non-canonical nucleic acid structures like G-quadruplex-forming RNA sequences, we analyzed the R-loop interactome for their ability to undergo LLPS to test the hypothesis that LLPS also plays a role in R-loop biology.

As described by Holehouse and Pappu (Holehouse and Pappu, 2018), an important framework for understanding biological LLPS is to think of proteins, nucleic acids and other biopolymers as multivalent associative polymers consisting of two or more interactive segments (“stickers”) that are linked by flexible linkers (“spacers”). For proteins, stickers could be folded domains (e.g., RRMs, KH domains) or short sequence motifs (e.g., RGG, SR motifs or even single amino acid residues) embedded within a longer IDR. The stickers mediate the transient attractive intermolecular interactions, while the spacers provide the flexibility and conformational heterogeneity required for LLPS. Indeed, LLPS is a cooperative, but non-stoichiometric, process mediated by the assembly of polymers *via* non-covalent physical cross-links. The strength of the cross-linking as well as the lifetimes over which these non-covalent cross-links occur will determine the physico-chemical properties of the resulting LLPS microenvironment and the nature of the emergent structural properties, which in turn, will determine whether the LLPS will result in functional biomolecular condensates or pathological aggregates in cells. Recent studies have provided numerous examples of LLPS-mediated condensates consisting of proteins (e.g., signaling puncta) (Li et al., 2012), nucleic acids (Jain and Vale, 2017) or heterogenous mixture of proteins and nucleic acids (e.g., stress granules, transcription factories) (Wheeler et al., 2016; Boija et al., 2018). Because of the modular domain architecture of the majority of the R-loop interactome proteins and modular nature of the R-loop structure per se (see above), it is highly likely that these proteins undergo heterogenous LLPS assembly with R-loops *via* multivalent synergistic interactions of the folded domains and IDRs within these R-loops interacting proteins (Martin et al., 2021). Therefore, we focused on analyzing the ability of the R-loop proteins to undergo LLPS or to localize to membraneless organelles.

We used two LLPS prediction programs, PScore (Pi-Pi) (Vernon et al., 2018) and catGRANULE (Bolognesi et al., 2016), to investigate the propensity of the R-loop interactome to phase separate. Previous studies from Vernon and colleagues have shown that long-range planar π:planar π contact propensity, given by a calculated PScore, can identify many known phase-separating proteins (Vernon et al., 2018). These planar π:planar π interactions are mediated by sp hybrid-forming atom that are found in amino acids containing aromatic (Tyr, Phe, Trp, and His), amide (Gln, Asn), carboxyl (Glu, Asp), or guanidinium (Arg) groups. Thus, enrichment of these “stacking” amino acids in IDRs can be used to predict planar π:planar π-mediated LLPS (Vernon et al., 2018). In contrast, the catGRANULE algorithm can predict the tendency for a protein to assemble into membraneless foci mediated by LLPS by considering the contributions of nucleic acid binding propensities and structural disorder. According to the catGRANULE analysis, 67% of the Readers are predicted to undergo LLPS, while for the Combined Interactome and Enzymes groups, it is only 30 and 31% respectively (Supplementary Table S1B). In contrast, the PScore (Pi-Pi) program predicted that 59% of the Readers, but only 21 and 17% of the Combined Interactome and Enzymes, respectively, can undergo LLPS (Supplementary Table S1C). Taken together, these data suggest that the Readers have a higher propensity to undergo LLPS when compared to the Enzymes and the Combined Interactome (Figure 5B,C). We also investigated the percentage of R-loop interactome proteins found in LLPS databases including PhaSePro (Mészáros et al., 2020), LLPSDB (Li et al., 2020), and DRLLPS (Ning et al., 2020). Although the size and comprehensiveness of the databases are quite different and may affect the results, in all cases, the Readers have the highest level of presence in these LLPS databases when compared to the Combined Interactome or the Enzymes groups (Supplementary Table S1D). Based on these results, and the fact that the R-loop structure itself also provides multiple opportunities for multivalent interactions, as well as the observation that most of the proteins in the R-loop interactome are modular, there is a high probability that R-loop foci can be mediated by LLPS assembly driven mainly by the Readers, with the Enzymes acting as co-scaffolds or clients (Figure 2).

### Analysis of Amino Acid Composition of IDRs of R-Loop Interacting Proteins

As discussed above, the majority of the R-loop interactome are modular proteins (Figure 1), containing long stretches of IDRs that do not form stable folded structure. Here, we investigate in detail the amino acid composition and physico-chemical properties of the residues in the IDRs of these R-loop proteins in order to gain insights about the types of molecular interactions that they can form. With this knowledge, we can then compare and contrast similarities and differences of the IDRs of R-loop proteins to the C-IDR of FMRP and determine whether there is a potential universal mechanism of R-loop recognition. Previous research have shown that in general, DNA, and RNA binding proteins are enriched in positively charged (Arg, Lys) and aromatic (Trp, Tyr, His, and Phe) residues, but are depleted in negatively charged (Glu, Asp) and proline residues in the interfaces of protein-DNA or protein-RNA complexes (Terribilini et al., 2006; Yesudhas et al., 2017; Zhang et al., 2018; Bartas et al., 2021). However, for non-canonical nucleic acid structures, there is a global enrichment for Gly, Arg, Glu, Asp, and Val in G-quadruplex binding proteins (Takahama and Oyoshi, 2013; Yagi et al., 2018; Ishiguro et al., 2020), enrichment for Lys and Ser in Cruciform binding proteins (Brázda et al., 2011; Bartas et al., 2019) and enrichment for Asn, Asp, Ile, and Tyr for Triplex binding proteins (Bartas et al., 2021). Therefore, we performed a detailed amino acid composition analysis to determine whether there exists global enrichment or depletion of certain amino acid composition or patterns required for R-loop interaction.

**FIGURE 6:**
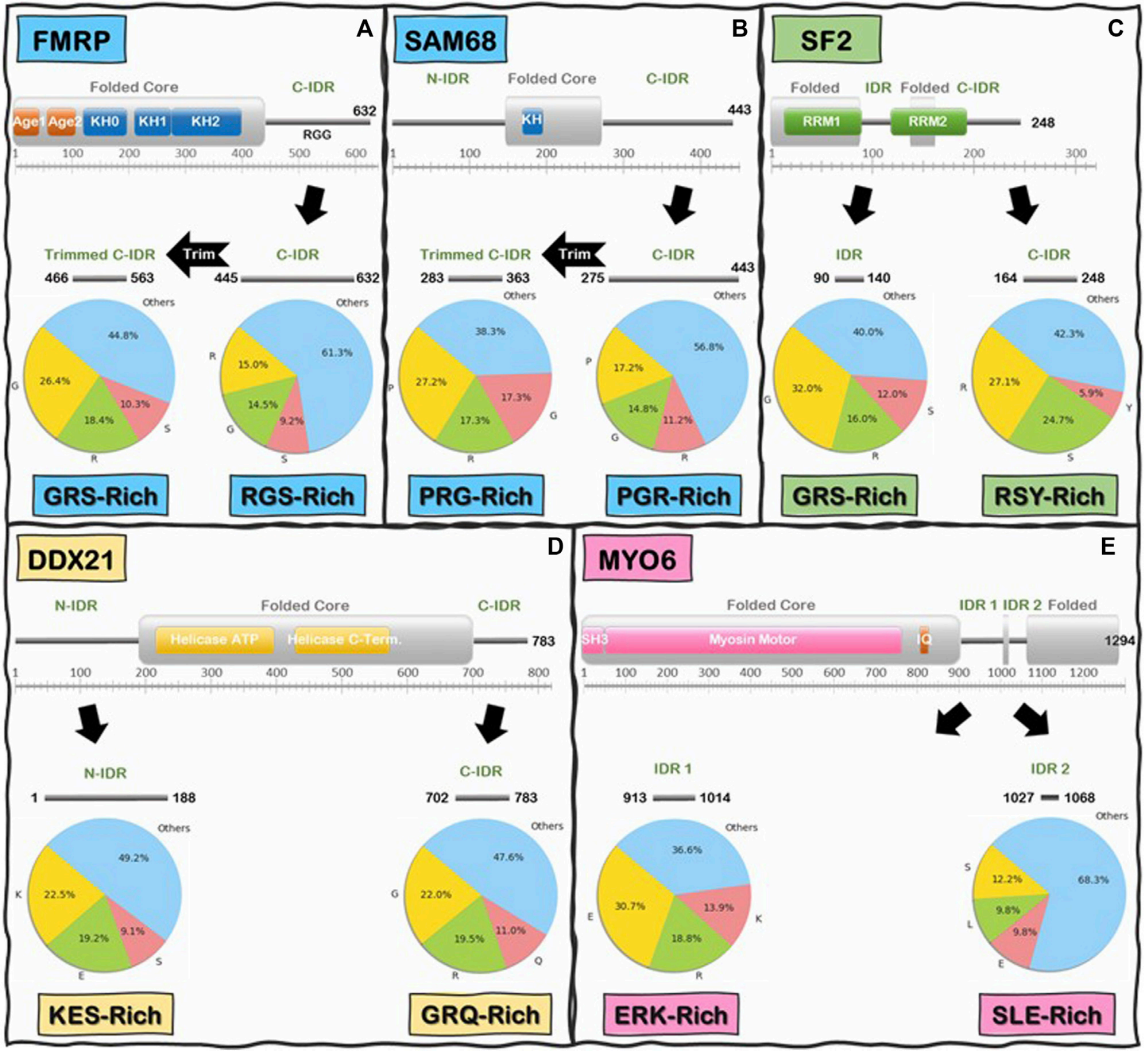
Schematics of low-complexity sequence in the IDRs of Readers and Enzymes of the R-loop Interactome. First and last residue number for each IDR is indicated along the IDR schematics. Pie charts indicate the frequency (%) of the three most abundant amino acids in the IDR above, and the remaining 17 amino acids are grouped into “Others”. Boxes underneath pie charts indicate, in decreasing order, the three most abundant amino acids in the IDRs above. **(A)** FMRP C-IDR’s RGS residues make up to 38.7% of the entire IDR, while this content increases to 55.2% in the subregion from residue 466 to residue 563. **(B)** SAM68 C-IDR’s PGR residues make up to 43.2% of the entire IDR, while this content increases to 61.7% in the subregion from residue 283 to residue 363. **(C)** SF2 internal IDR’s GRS residues make up to 60% of the entire IDR content while C-IDR’s RSY residues make up to 57.7% of the entire IDR content. **(D)** DDX21 N-IDR’s KES residues make up to 50.8% of the entire IDR content while C-IDR’s GRQ residues make up to 52.4% of the entire IDR content. **(E)** MYO6 internal IDR 1’s ERK residues make up to 63.4% of the entire IDR content while internal IDR 2’s SLE residues make up to 31.7% of the entire IDR content. Interestingly, the IDRs of Readers and Enzymes are generally biased towards different residues.

We began by analyzing the frequencies of individual amino acids and we immediately noticed that, like the C-IDR of FMRP, these IDRs are highly enriched in low complexity (LC) sequences that are biased towards very few amino acids (Figure 6). In fact, for the majority of IDRs, especially in the Readers, a mere two to five different amino acids make up at least 50% or more of the total number of IDR residues, with the most prevalent amino acids being Glu, Ser, Lys, Pro, Gly, Ala, and Arg, and a noticeable depletion of aromatic residues (i.e., His, Phe, Tyr, and Trp) (Supplementary Table S2A), except for some R-loop Readers like SF2 and NONO, whose C-IDR and N-IDR are enriched in aromatic residues, respectively. Furthermore, these biased amino acid compositions are not uniformly distributed throughout the IDRs. Rather, certain segments of some IDRs are more “biased” than others (Figure 6). For example, Arg, Gly, and Ser make up 38.7% of the FMRP’s C-IDR, however this value goes up to 55.2% for the region from residues 466 to 563, and for the region from residue 527 to 552, just Arg and Gly alone make up 83% of the sequence (Figure 6A). In the case of the C-IDR of SAM68, Pro, Gly, and Arg make up 43.2% of the entire IDR, but for the region (283–363), these residues make up 61.7% (Figure 6B). As discussed below, it will be quite interesting to determine whether the heavily biased low complexity segments 1) form the binding motifs that interact with R-loops, 2) form stickers to drive LLPS, or 3) perform other biological functions, such as recruitment or binding other factors.

Interestingly, when we compared the Reader and the Enzymes, we found that there is a significant differential biased amino acid composition between the IDRs of R-loop Readers and Enzymes. On average, Readers are enriched in Gly, Ser, Arg, and Pro residues while the Enzymes are enriched in Glu, Lys, Arg, and Ser (Supplementary Table S2A). For instance, in the C-IDR of SF2, Arg, and Ser residues alone make up approximately 51.8% of this IDR (Figure 6C) and, as mentioned above, in the RGG domain (residue 527–552) of the C-IDR of FMRP, Arg, and Gly alone make up 83% of the sequence. In contrast, for the enzyme DDX21, Lys, Glu, and Ser makes up to 50.8% of the entire N-terminal IDR (N-IDR), while Gly, Arg, and Gln make up to 52.4% of the C-IDR (Figure 6D). Similarly, for the case of the enzyme Myosin VI (Vreugde et al., 2006), Glu, Arg, and Lys, and Ser, Leu, and Glu, make 63.4 and 31.7% of the IDR1 and IDR2, respectively (Figure 6E).

Furthermore, while the biased low complexity sequences in the different IDRs are not identical in terms of individual amino acids constituents, they are noticeably similar in terms of physico-chemical properties of the enriched amino acids. For example, the high content of Arg and Gly in the C-IDR of FMRP is quite similar to the Arg and Ser composition in the C-IDR of SF2 when we consider that Gly and Ser have very similar properties (e.g., being small and flexible). Therefore, to explore the amino acid composition similarities further, we performed a reduced amino acid alphabet clustering (Murphy et al., 2000; Weathers et al., 2004; Peterson et al., 2009), where the 20 standard amino acids are classified, based on relatively similar physico-chemical properties, into six groups (Supplementary Figure S1): 1-Aromatic (Tyr, Trp, and Phe); 2-Positively Charged (Arg, Lys, and His); 3-Polar, uncharged (Asn, Gln); 4-Negatively Charged (Asp, Glu); 5-Small/Flexible (Gly, Ser, Pro, Ala, Thr, and Cys); and 6-Hydrophobic (Ile, Leu, Val, and Met). Our analysis shows that Groups 2 and 5 are the most prevalent (Supplementary Table S2B), and more interestingly, when we analyze the frequencies of 2-mer (i.e., dipeptides) occurrences along the IDR sequences of the R-loop proteins, we found that almost a third (∼35%) of 2-mers in all the IDRs investigated are a group 5 or 2 member followed by another group 5 or 2 member (Supplementary Table S3B) with GG, PP, RS, SR, and RG, being the most frequent 2-mer for the R-loop Readers, while for the Enzymes, it is EE, KK, KE, EK, and GG (Supplementary Table S3A). Furthermore, when we analyzed the 3-mer (i.e., tripeptide) frequencies, we found the most striking differences between Readers and Enzymes. For instance, the most prevalent tripeptides are GGG, SRS, RSR, PPP, and GRG for the Readers, but EEE, GGG, KKK, KEE, and EKE for the Enzymes (Supplementary Table S4A shows the top ten 3-mers found in the R-loop interactome). Therefore, these analyses reveal that emergent molecular features (charge patterning and flexibility) show marked similarities and differences within and between the IDRs of Readers and Enzymes of the R-loop interactome, respectively. Furthermore, our analysis of the amino acid composition of these IDRs reveal that they contain the amino acids necessary for LLPS and for non-canonical nucleic acid binding (vide supra), thus explaining how R-loop Readers may simultaneously undergo LLPS and interact with R-loops as we have demonstrated for FMRP (Tsang et al., 2019; Chakraborty et al., 2021). A fundamental question that needs to be experimentally answered is whether these observed differences in these IDRs translate into differences in R-loop binding affinity or LLPS foci assembly.

## Discussion

The overarching goal of this work is to explore the modular domain architecture and phase separation propensity of the R-loop interactome as well as to investigate the physico-chemical properties of the amino acids and the emergent molecular features within the IDRs of these proteins. Here, we present a provoking hypothesis that these IDRs could indeed be the predominant sites for interaction with R-loops, as we recently discovered for the C-IDR of FMRP. While IDRs normally function as linkers connecting folded domains of proteins or as sites of PTMs for regulating the function of folded domains, IDRs of proteins can also be the main site of biological activity, as it was recently demonstrated for the intrinsically disordered protein 4E-BP2 (Bah et al., 2015; Bouvignies and Blackledge, 2015; Dawson et al., 2020). In fact, for DNA or RNA G-quadruplexes, which are another major type of non-canonical nucleic acid structures, it is well-documented that, in many cases, it is the IDRs that are mainly involved in making direct contacts (Vasilyev et al., 2015; Huang et al., 2018). Furthermore, it has been demonstrated that G-quadruplexes can trigger LLPS (Zhang et al., 2019). So, the question is whether a similar mechanism occurs between IDRs and R-loops as well. Indeed, we observe a significant overlap between the R-loop and G-quadruplex interacting proteins, including FMRP (Supplementary Table S1E). In the case of FMRP, the RGG-rich region within the C-IDR of FMRP binds to multiple segments of the G-quadruplex structure including the duplex–quadruplex junction, the mixed tetrad, and the duplex region of the RNA *via* cation–π interactions, shape complementarity, and multiple hydrogen bonds (Vasilyev et al., 2015). The structural mechanism by which the C-IDR of FMRP or the IDRs in other R-loop binding proteins interact with R-loops is currently unknown, but this mechanism is being intensely investigated in our laboratory. It will be quite interesting to determine whether it is the same G-quadruplex-binding RGG-rich region or a different segment of the C-IDR of FMRP that binds the R-loop. Indeed, the *in vivo* formation of R-loops and DNA G-quadruplexes are intimately coupled during transcription, and the fact that we observed that many (32 proteins) of the R-loop proteins are also G-quadruplexes binding proteins further re-enforces the linkage between the biological functions of these two non-canonical nucleic structures (Kuznetsov et al., 2018; Maffia et al., 2020). Determining the biological mechanisms utilized to control the differential or simultaneous interactions of these overlapping proteins with these two distinct non-canonical nucleic acid structures will be fascinating to explore.

Another critical question waiting to be answered is whether there is a difference in the mechanism of R-loop binding between the subclasses of IDRs found in the R-loop Readers vs. R-loop Enzymes. Our observation that Readers are enriched in Arg, Ser, Gly, and Pro-containing motifs, while Enzymes contain Glu, Lys, and Gly-containing motifs raises an important question about the potential differential mechanism of IDR-mediated R-loop interaction or phase separated assembly. Indeed, a recent seminal paper by Fisher and Elbaum-Garfinkle demonstrated that poly-Arg: and poly-Lys:nucleic acid condensates form distinct and immiscible phase separated droplets. However, how the introduction of other amino acids, such as Ser, Gly, and Pro into the Arg-rich IDRs observed in Readers compared to the Glu and Gly into the Lys-rich Enzyme IDRs, affect their phase separation behavior will need to be empirically tested. Our hypothesis that IDRs of R-loop binding proteins can potentially bind to and co-phase separate with their R-loop substrates provides another exquisite example of the versatility of IDRs of proteins in utilizing identical or similar array of molecular features to mediate diverse biological processes. Taken together, our analysis suggests that the modular R-loop interacting proteins can utilize the synergy of their folded domains and their IDRs to engage in multiple, dynamic interactions with R-loops and various R-loop-resolving factors (*i.e.*, helicases, nucleases, topoisomerases, *etc.*) to assemble a conducive biochemical, membraneless microenvironment to recognize and resolve unscheduled R-loops in a timely manner (Figure 2). As the potential of IDRs as therapeutics are being increasingly realized, the study of the mechanisms for R-loop recognition and phase separation by IDRs is timely and will undoubtedly open many avenues for the development of novel therapeutics for cancers and neurological diseases that are mediated by the dysregulation of R-loop function (Wang et al., 2011; Uversky, 2012; Ambadipudi and Zweckstetter, 2016; Wheeler, 2020).

## Methods

### Bioinformatics Analysis and Data Processing

Canonical protein sequences of all the proteins used in this study were downloaded from the UniProt database (UniProt: The universal protein knowledgebase in 2021, 2021). Prediction of the modular domain architecture of the R-loop proteins (i.e., fold/disorder organization) was conducted with the aid of the program IUPRED2A web server (Erdős and Dosztányi, 2020). Predictions of liquid-liquid phase separation behavior were conducted with the programs catGRANULE (Bolognesi et al., 2016) (web server) and PScore (Vernon et al., 2018) (downloadable version). All programs were operated using their default parameters. Sequence composition studies were conducted using in-house Python scripts, which are available upon request. The data was compiled, processed and analyzed using Python scripts and Microsoft Excel Spreadsheets generated in our lab. Statistical analyses to compare average prediction scores between groups consist of one-way ANOVA followed by Tukey-Kramer post-hoc test for all pairwise combinations (*p* < 0.05) conducted on Microsoft Excel. Uniprot ID, gene name, and prediction scores for the proteins from the R-loop interactome are compiled into Supplementary Table S5. Uniprot ID for the proteins from the G-quadruplex (G4) database and the LLPS databases are compiled into Supplementary Tables S6, S7, respectively.

## Data Availability

The datasets presented in this study can be found in online repositories. The names of the repository/repositories and accession number(s) can be found in the article/Supplementary Material.
