# Supplementary figures and images for: A Tale of Loops and Tails: The Role of Intrinsically Disordered Protein Regions in R-Loop Recognition and Phase Separation
Source: Front Mol Biosci. 2021 Jun 10;8:691694. doi: 10.3389/fmolb.2021.691694 (PMC8222781; doi:10.3389/fmolb.2021.691694)

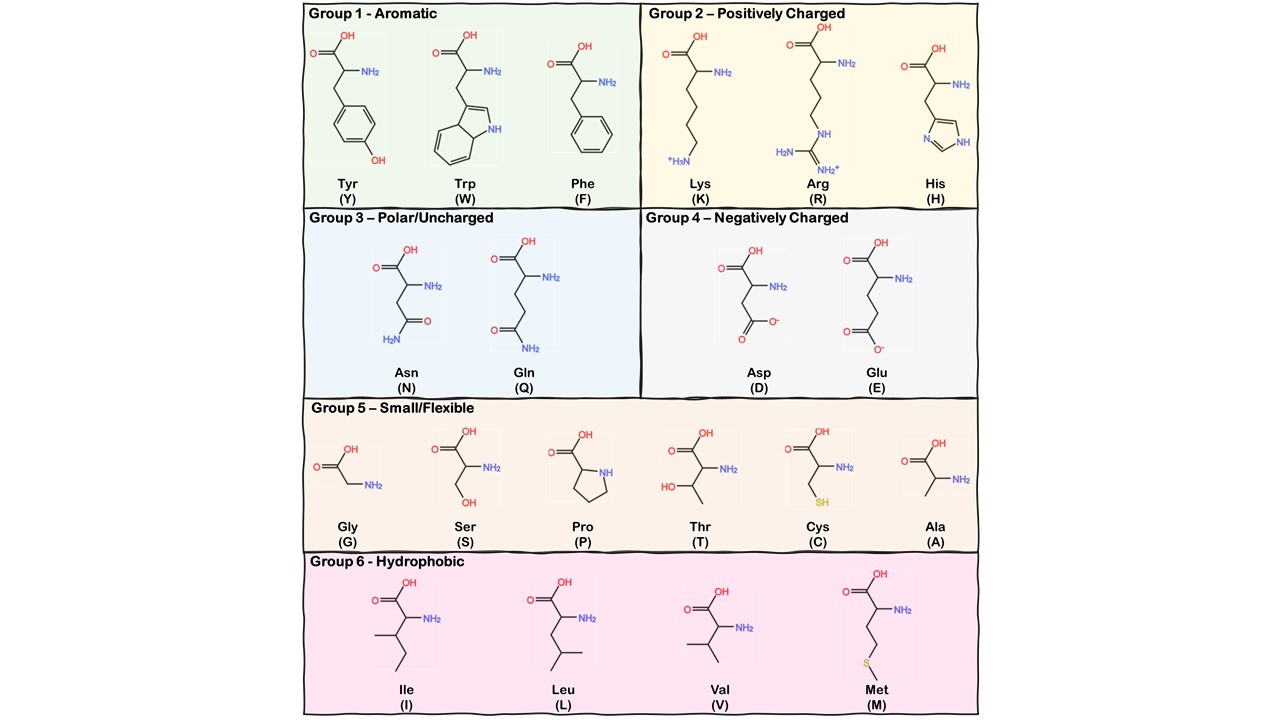

Supplement: Supplementary file 3 [file Image1.JPEG]
